# Evolution and characteristics of tetracycline resistance in *Mycoplasma pneumoniae*

**DOI:** 10.1128/spectrum.03398-24

**Published:** 2025-05-23

**Authors:** Panpan Xie, Jie Liu, Yuzhong Feng, Taiyun Zhao, Wenkai Niu, Yun Fang, Xin Zhang, Xilin Zhao, Fusheng Wang, Xin Yuan

**Affiliations:** 1 Department of Respiratory and Critical Care Medicine, Senior Department of Infectious Diseases, The Fifth Medical Center of PLA General Hospital, Beijing, China; 2 Senior Department of Infectious Diseases, The Fifth Medical Center of PLA General Hospital, National Clinical Research Center for Infectious Diseases, Beijing, China; 3 Anhui Medical University 12485, Hefei, Anhui, China; 4 Department of Intensive Care Unit, Hainan Hospital of PLA General Hospital, Sanya, China; 5 Academy of Military Medical Sciences, Academy of Military Sciences, Beijing, China; 6 Institute of Pharmacology and Toxicology, Academy of Military Medical Sciences 71040, Beijing, China; 7 Public Health Research Institute and Department of Microbiology, Biochemistry and Molecular Genetics, New Jersey Medical School, Rutgers Biomedical and Health Sciences, Rutgers University, Newark, New Jersey, USA; 8 State Key Laboratory of Molecular Vaccinology and Molecular Diagnostics, School of Public Health, Xiamen University 554989, Xiamen, China; Petrified Bugs LLC, Miami, Florida, USA

**Keywords:** *Mycoplasma pneumoniae*, tetracycline resistance, inducible resistance, point mutation, reduced ribosomal affinity, efflux pump

## Abstract

Macrolide resistance in *Mycoplasma pneumoniae* is becoming prevalent. Tetracyclines are an important alternative for patients with macrolide-resistant *M. pneumoniae* infections. Tetracycline-resistant *M. pneumoniae* has never been reported in clinical isolates or *in vitro*-induced strains. Here, we aimed to explore tetracycline-resistant *M. pneumoniae in vitro* and the underlying resistance mechanisms. Clinically isolated parental *M. pneumoniae* strains were selected for tetracycline resistance by inducing them *in vitro* with increasing concentrations of tetracycline, minocycline, and tigecycline. The whole genomes of the induced resistant and parent strains were both sequenced to identify tetracycline-resistance genes, and their 16S rRNA gene sequences were compared. Ribosome-binding assays were conducted by incubating the 30S ribosomes of the resistant and sensitive strains with various concentrations of ^3^H-labeled tetracycline. The effect of efflux pump inhibitor reserpine on the susceptibility of tetracyclines to the resistant mutants was evaluated. Eight resistant mutants were obtained from 12 tetracycline-susceptible *M. pneumoniae* strains. Whole-genome sequencing identified point mutations in the 16S rRNA gene of all the resistant mutants. The ribosomes of two induced resistant strains showed lower tetracycline-binding capacities than their parental strains. Reserpine increased the susceptibility of two resistant mutants to tetracyclines by more than fourfold. None of the resistant mutants carried the known tetracycline-resistance genes. *M. pneumoniae* strains can develop tetracycline resistance. Mutations in the 16S rRNA gene leading to decreased ribosomal-binding capacity and the presence of efflux pumps may be the mechanisms associated with tetracycline resistance in *M. pneumoniae*. This study identifies markers to track the emergence of tetracycline resistance in *M. pneumoniae*.

**IMPORTANCE:**

Resistance to anti-*Mycoplasma pneumoniae* antibiotics is a global public health problem. Effective antibiotic treatment strategies are urgently needed. Tetracyclines are among the most commonly used antibiotics for the treatment of drug-resistant *M. pneumoniae* infections. Tetracycline-resistant *M. pneumoniae* has not been reported; however, treatment with increased concentrations of tetracycline selects hyposensitive mutants. In this study, we successfully induced tetracycline-resistant *M. pneumoniae* mutants by 10-month *in vitro* serial passage experiments and found that a 16S rRNA mutation resulting in reduced ribosomal drug binding was the mechanism that led to tetracycline resistance in *M. pneumoniae*. In addition, the efflux pump might also be related to decreased tetracycline susceptibility. This study presented the first data on the induction of tetracycline resistance in *M. pneumoniae in vitro*, providing insights into the development of effective treatment strategies for drug-resistant *M. pneumoniae* infections in the future.

## INTRODUCTION

*Mycoplasma pneumoniae* is a leading cause of respiratory infections in humans. It is estimated to be accountable for 10%–30% of community-acquired pneumonia (CAP) cases, posing critical clinical challenges worldwide (1, 2). *M. pneumoniae* is an atypical pathogen as it lacks a cell wall, rendering it resistant to a wide range of antimicrobial drugs that target the cell wall, such as β-lactams (3, 4). Three classes of antimicrobial drugs have demonstrated efficacy against *M. pneumoniae*: fluoroquinolones, which inhibit DNA replication, and macrolides and tetracyclines, which inhibit protein synthesis (4). However, a growing body of research has reported increasing macrolide resistance in *M. pneumoniae* worldwide, particularly in China and Japan, where over 90% of *M. pneumoniae* isolates exhibit resistance to macrolides (4–6). Quinolones are not recommended for patients below 18 years due to the risk of cartilage damage (7). The quinolone-resistant *M. pneumoniae* strains were selected *in vitro,* and this resistance was attributed to mutations in the *gyrA*, *gyrB*, *parC*, and *parE* genes (8). Furthermore, Yuan et al. reported a case in which fluoroquinolone was ineffective in treating an adult with *M. pneumoniae* infection (9). Given this rise in macrolide resistance, tetracyclines are now the only viable treatment option for drug-resistant *M. pneumoniae* infections in patients aged 8–18. Furthermore, tetracyclines are crucial drug candidates for treating adults infected with *M. pneumoniae* strains that are resistant to macrolides (10). Although tetracycline resistance has not been reported in *M. pneumoniae*, the rise in tetracycline prescriptions makes monitoring and preventing resistance to this antimicrobial agent crucial.

Bacteria lose their susceptibility to tetracyclines via four main mechanisms: efflux, ribosomal protection, enzymatic inactivation of tetracyclines, and rRNA gene mutations (11–13). According to a tetracycline resistance gene database, 37 tetracycline-specific efflux pump genes, 13 ribosomal protection genes, 13 enzymatic genes, 1 gene of unknown classification, and 11 mosaic ribosomal protection genes have been identified to date (https://faculty.washington.edu/marilynr/tetweb1.pdf). The *tetM* gene, which confers resistance to tetracyclines, has been identified in *Mycoplasma hominis* and *Ureaplasma urealyticum* (14, 15). Tetracycline resistance in *Streptococcus pneumoniae,* another leading cause of CAP, has been attributed to *tetM*- and *tetO*-mediated ribosomal protection, the mosaic *tetS/M* gene (16), and mutations in the coding sequences of ribosomal protein S10 and the ABC-type transporter PatA (17). *M. pneumoniae* and *S. pneumoniae* co-infections are common. In such cases, it remains unclear whether tetracycline-resistance genes are transferred from *S. pneumoniae* to *M. pneumoniae* through plasmids or other mobile genetic elements, conferring tetracycline resistance to *M. pneumoniae*. Dégrange et al. developed reduced-susceptibility mutants of *M. pneumoniae in vitro*, with a minimum inhibitory concentration (MIC) of up to 2 mg/L for tetracycline, and identified 16S rRNA gene mutations in these mutant strains (11). However, tetracycline resistance in *M. pneumoniae* has not been researched to date.

This study aims to understand the mechanism of tetracycline resistance in *M. pneumoniae* and enhance the clinical utility of tetracyclines. To this effect, we developed tetracycline-resistant *M. pneumoniae* mutants by exposing parent strains to increasing concentrations of three tetracyclines *in vitro*. Whole-genome sequencing, ribosome-binding assays, and efflux pump inhibition assays were performed to investigate the resistance mechanism in these mutants.

## MATERIALS AND METHODS

### Isolation and antimicrobial susceptibility testing of *M. pneumoniae* strains

Clinical isolates of *M. pneumoniae* were obtained from oropharyngeal and sputum samples collected from patients treated for lower respiratory infections at our hospital. Preliminary identification of the isolates was based on real-time polymerase chain reaction (PCR), which was then confirmed by colony morphology (18, 19). The susceptibilities of the *M. pneumoniae* isolates to tetracycline, minocycline, and tigecycline were tested using the broth microdilution method as recommended by the Clinical and Laboratory Standards Institute (CLSI, document M43A) (20). Tetracycline and minocycline standards were purchased from the National Institute for the Control of Pharmaceutical and Biological Products, while tigecycline standards were purchased from Cato Research Chemicals Inc., USA. MIC is defined as the lowest concentration of an antimicrobial agent inhibiting visible color change in the culture medium during a 14-day incubation at 37°C; for tetracycline, an MIC of 2 mg/L or lower indicates that the test organism is sensitive to this antibiotic (20). *M. pneumoniae* reference strain FH (ATCC15531, USA) was used as the quality control. Due to the lack of tetracycline-resistant *M. pneumonia*e strains, the CLSI document M43A does not define any MIC categories (intermediate or resistant categories) for tetracyclines besides “susceptible.” The resistance breakpoints used in this study were determined based on relevant literature and the resistance criteria for Gram-positive bacteria and *M. hominis* (MICs ≥ 8 mg/L) detailed in CLSI M100 Ed-29 and CLSI-M43A (11, 20–22), respectively. For tetracycline, minocycline, and tigecycline, MICs ≥ 8 mg/L were classified as resistant to *M. pneumonia*e in this study.

### Selection of tetracycline-resistant mutant strains of *M. pneumoniae*

The *in vitro* selection of tetracycline-resistant mutants was conducted as described previously, with minor modifications (11, 23). In brief, *M. pneumoniae* was inoculated into the induction medium with each of the three tetracyclines at four different concentrations (1/4 × MIC, 1/2 × MIC, 1 × MIC, and 2 × MIC), which were determined according to the MICs for each *M. pneumoniae* strain. The strains were incubated at 37°C for 14 days. The highest concentration at which *M. pneumoniae* could grow was selected, and the *M. pneumoniae* strains in this concentration of culture medium were selected for the next passaging. For subsequent passages, half of the highest tolerated antibiotic concentration was used as the new starting concentration. The selected strains were transferred to fresh media containing four new antibiotic concentrations (adjusted accordingly) and cultured under the same conditions. This process was repeated to gradually escalate antibiotic pressure, with the starting concentration doubled every two passages. The induction continued until *M. pneumoniae* strains demonstrated stable growth in media containing tetracycline antibiotics at a concentration of 4 mg/L. At this stage, the MIC values of the strains for three tetracyclines were re-evaluated. Strains with MIC ≥ 8 mg/L were classified as successfully induced resistant strains. If MIC remained < 8 mg/L, additional passages were performed, followed by repeated MIC validation. The parent strains were continuously passaged 14 times without the addition of antibiotics, and the time between each passage was the same as that of the resistance induction assay.

### Whole-genome sequencing

Genomic DNA was extracted from a 9 mL broth culture of the induced resistant and parent strains of *M. pneumoniae* using a QIAamp DNA mini kit (QIAGEN, Germany) and subjected to high-throughput sequencing on an Illumina MiSeq platform (San Diego, CA, USA). Libraries with an insert size of 600 bp were constructed using the MiSeq Reagent Kit v2. Sequence data were assembled using Newbler v2.9. The assembled whole genomes of the strains were submitted to NCBI’s GenBank and annotated using NCBI’s Prokaryotic Genome Annotation Pipeline. The 16S rRNA gene sequences of the resistant and sensitive strains were compared on CLC Genomics Workbench 3, a genomics data analysis software. On the Center for Genomic Epidemiology website (http://www.genomicepidemiology.org/), we uploaded the whole-genome sequences of 8 induced tetracycline-resistant strains to ResFinder version 2.1 to identify acquired genes and/or find chromosomal mutations mediating antimicrobial resistance in the total or partial DNA sequence of bacteria. The analysis parameters were configured as follows: the threshold for resistance gene identification was set at 70% identity, minimum length coverage at 60%, and the relevant antibiotic was specified as tetracycline. The input file format was selected as assembled sequences. Following software execution, the screening results were automatically fed back to the interface.

### Ribosome-binding assay

Ribosomes from two representative resistant strains and the corresponding sensitive parent strains were extracted as described in a previous study (23). The representative resistant strains were selected based on the greatest degree of change in MIC observed before and after the induced resistance assay. The ribosomal affinity of tetracycline in a representative drug-resistant strain and its corresponding drug-sensitive parent strain was compared using ^3^H-labeled tetracycline with modifications to a previously described protocol (23). For the binding assay, 300 µL samples containing a fixed amount of ribosome (A_260_ = 0.1) and varying quantities of ^3^H-labeled tetracycline (100 pmol, 50 pmol, 25 pmol, 12.5 pmol, 6.25 pmol, and 3.125 pmol; ART 0239A, 1 mCi, Tongfu Company, China) were incubated at 22°C for 30 min. Repeat twice for each isotope concentration. The reaction was stopped by adding 5 mL of reaction termination solution Buffer B (10 mM Tris, 5 mM MgCl_2_, and 150 mM KCl at pH 7.2). The reaction solutions were passed through filter membranes, which were subsequently washed 2–3 times with TMMKA-100 buffer. The washed membranes were carefully removed, dried, and soaked in 1 mL of scintillation fluid. Radioactive count per minute (CPM) was measured using a liquid scintillation analyzer (Tri-carb 9100, PerkinElmer Company, USA). The CPM represents the amount of tetracycline bound to the ribosomes. The differences between the ribosomal affinity of tetracycline in the sensitive and resistant strains were determined by comparing their radioactive CPM.

### Efflux pump inhibition assay

The efflux pump inhibitor reserpine was obtained from the National Institute for the Control of Pharmaceutical and Biological Products. The MICs of tetracycline for the resistant and reference (FH) *M. pneumoniae* strains were determined as described previously (24). MICs were determined under four conditions: in the presence of *M. pneumoniae* strains alone, tetracycline alone, reserpine alone (20 mg/L), and a combination of tetracycline and reserpine (20 mg/L).

## RESULTS

### *M. pneumoniae* isolates are susceptible to three types of tetracyclines

We isolated and identified 31 *M*. *pneumoniae* strains using culture and real-time PCR methods. The MIC_50_ and MIC_90_ of tetracycline, minocycline, and tigecycline for the reference strain FH were both 0.5 mg/L. The MIC_50_ and MIC_90_ of tetracycline of the 31 *M*. *pneumoniae* strains were 0.5 mg/L and 1 mg/L, respectively. The corresponding values were 0.5 mg/L and 0.5 mg/L for minocycline and 0.25 mg/L and 0.5 mg/L for tigecycline.

### Selection of tetracycline-resistant mutants

After the continuous passage without the addition of antibiotics, the subcultures showed no change in MICs, indicating that passage pressure has no effect on mutation occurrence. Twelve tetracycline-susceptible *M. pneumoniae* strains were treated with tetracycline, minocycline, and tigecycline *in vitro*. Eight resistant *M. pneumoniae* strains were obtained after serial passages for 10 months. The MICs of five induced strains to tetracycline and minocycline were determined to be ≥ 8 mg/L at the 12th passage, while the remaining three induced strains exhibited MIC values > 8 mg/L at the 14th passage. Although no tigecycline-resistant strains could be induced even after 14 passages, the MIC of tigecycline increased to 2–4 mg/L.

The selected resistant mutants displayed different MIC characteristics for the three tetracyclines. At the 12th passage, three minocycline-resistant mutants (including FH-tet-R, which evolved from the reference strain FH) were obtained. Furthermore, the MICs for the three tetracyclines increased 16- to 64-fold for tetracycline, 16- to 32-fold for minocycline, and 4- to 8-fold for tigecycline. Two tetracycline-resistant mutants were obtained with increases in the MICs of 16- to 32-fold for tetracycline, 16-fold for minocycline, and 8-fold for tigecycline. At the 14th passage, one minocycline-resistant mutant was obtained with increases in the MICs of 32-fold for tetracycline, 16-fold for minocycline, and 8-fold for tigecycline. In addition, two tetracycline-resistant mutants were obtained, and the MICs of the three tetracyclines increased (32-fold for tetracycline, 8- to 16-fold for minocycline, and 8- to 32-fold for tigecycline). Details of the eight tetracycline-resistant mutants are shown in Table 1.

**TABLE 1:** MIC changes before and after serial passages^*a*^

*M. pneumoniae* strain	Type and concentration (mg/L) of drug	MIC (mg/L)		
		Tetracycline	Minocycline	Tigecycline
FH-tet-R	Minocycline 4	0.5→8	0.5→16	0.5→4
S34-tet-R	Minocycline 4	0.25→16	0.125→4	0.25→2
S4-tet-R	Minocycline 4	0.5→8	0.5→8	0.5→2
S55-tet-R	Minocycline 8	0.5→16	0.5→8	0.25→2
S68-tet-R	Tetracycline 4	0.5→16	0.5→8	0.25→2
S12-tet-R	Tetracycline 4	0.5→8	0.5→4	0.25→2
S91-tet-R	Tetracycline 8	0.5→16	0.5→4	0.25→2
S63-tet-R	Tetracycline 8	0.5→16	0.5→8	0.125→4
FH control	Tetracycline/minocycline/tigecycline 8	0.5→0.5	0.5→0.5	0.5→0.5

^*a*^ The → symbol indicates changes in the MIC before and after serial passages with increasing concentrations of tetracyclines.

### Analysis of the whole genomes of the selected mutants

The whole genomes of the eight selected resistant mutants and their sensitive parent strains were sequenced, and the genome sequences of the tetracycline-resistant strains were submitted to the NCBI GenBank database. The genomes were annotated using NCBI’s Prokaryotic Genome Annotation Pipeline. The genomes of the eight strains were 99.9% identical.

**Fig 1:**
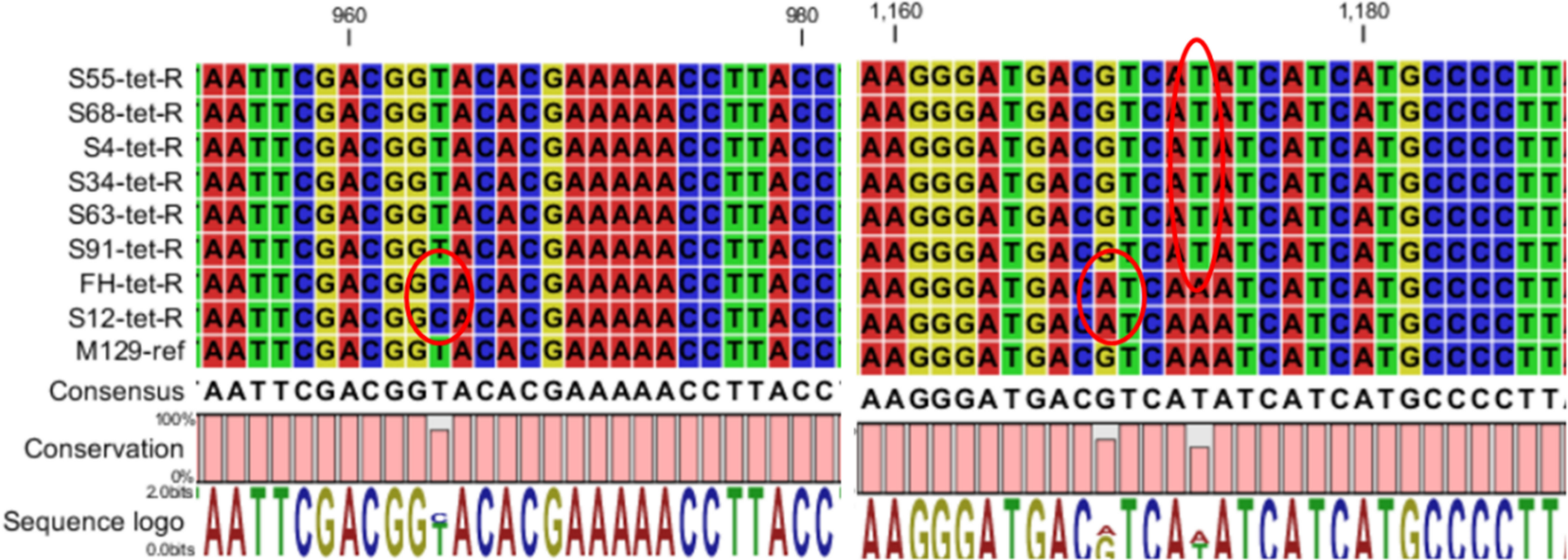
Mutations detected in the 16S rRNA gene sequences of the tetracycline-resistant *Mycoplasma pneumoniae* strains. The 16S rRNA gene sequences of the eight resistant mutants and the reference strain (M129) were aligned. The mutated bases are circled in red, and the consensus sequence and degree of conservation are displayed below the alignment.

The 16S rRNA gene sequences of the eight tetracycline-resistant strains were compared with those of the corresponding parent strains and *M. pneumoniae* reference strain M129 (GenBank accession no. CP003913). Both the susceptible and resistant strains displayed A71G point mutations. Six drug-resistant strains carried an A1173T mutation, while the other two strains exhibited two mutations: T964C and G1169A (summarized in Fig. 1 and Table 2).

**TABLE 2:** Characteristics of the assembled genomes of the eight tetracycline-resistant *M. pneumoniae* strains

Strain	Accession number	Length	% GC	No. of genes				Point mutations in the 16S rRNA gene
				CDS^*a*^	rRNA	tRNA	Total	
FH-tet-R	CP020690	816378	0.400	747	6	37	790	A71G, T964C, G1169A
S68-tet-R	CP020691	816404	0.400	749	6	37	792	A71G, A1173T
S55-tet-R	CP020692	815909	0.400	747	6	37	790	A71G, A1173T
S91-tet-R	CP020693	816429	0.400	748	6	37	791	A71G, A1173T
S63-tet-R	CP020689	816435	0.400	749	6	37	792	A71G, A1173T
S4-tet-R	CP020711	816433	0.400	748	6	37	791	A71G, A1173T
S12-tet-R	CP020712	816430	0.400	747	6	37	790	A71G, T964C, G1169A
S34-tet-R	CP020710	816433	0.400	748	6	37	791	A71G, A1173T

^*a*^ CDS: coding sequences.

The whole genomes of the eight tetracycline-resistant strains were uploaded to Resfinder 2.1 (http://www.genomicepidemiology.org/) to identify resistance genes. The ID threshold and minimum length were set to 70% and 60%, respectively. No *tetM* or other tetracycline resistance genes were identified.

### Ribosomal affinity of the tetracycline-resistant mutants

**Fig 2:**
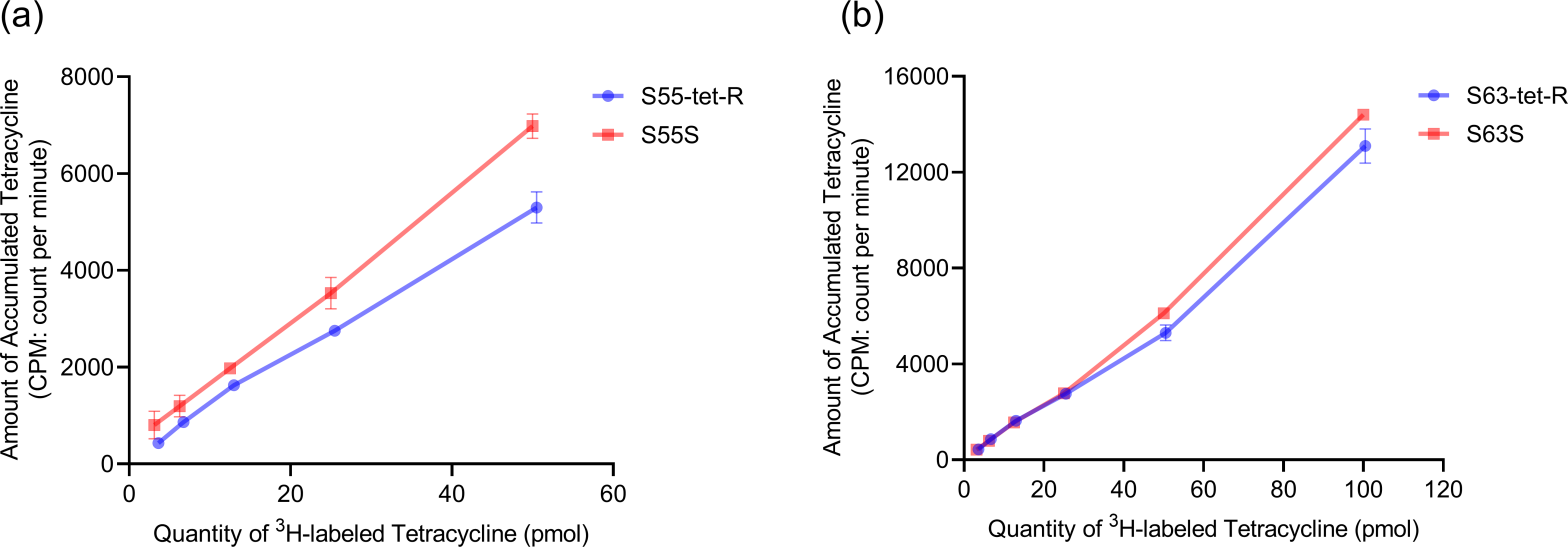
(**a**) Binding of ^3^H-labeled tetracycline to ribosomes from the resistant *M. pneumoniae* strain S55-tet-R and its susceptible parent strain S55S. (**b**) Binding of ^3^H-labeled tetracycline to ribosomes from the resistant *M. pneumoniae* strain S63-tet-R and its susceptible parent strain S63S. The data are presented as the arithmetic mean of two parallel replicates. Error bars represent the range of values.

Ribosome-binding assays for tetracycline were performed with two resistant strains and their corresponding susceptible parent strains. The tetracycline-binding capacity of the ribosomes in the induced resistant mutants was lower than that in the susceptible strains before induction (Fig. 2). This indicates that reduced ribosomal affinity may contribute to tetracycline resistance in *M. pneumoniae*.

### Efflux pump mechanism in tetracycline-resistant mutants

To assess the role of efflux pumps in tetracycline resistance, MICs were determined in the presence and absence of reserpine, an efflux pump inhibitor. Reserpine (20 mg/L) did not affect the growth of *M. pneumoniae* or the MIC of tetracycline for the reference strain FH (Table 3). However, reserpine reduced the MIC of tetracycline for two drug-resistant strains by more than fourfold, while exhibiting minimal or no influence on the MIC for other resistant strains (Table 3).

**TABLE 3:** Effect of reserpine on the MICs of tetracyclines for the tetracycline-resistant *M. pneumoniae* strains^*a*^

*M. pneumoniae* strain	MIC (mg/L)		
	Tetracycline only → with reserpine	Minocycline only → with reserpine	Tigecycline only → with reserpine
S68-tet-R	8→ 2	4→0.5	1→ 0.125
S55-tet-R	16→ <0.25	8→4	2→ 1
S91-tet-R	8→8	8→4	2→1
S63-tet-R	16→8	8→8	4→4
S4-tet-R	8→8	8→8	2→2
S12-tet-R	8→8	8→4	2→2
S34-tet-R	16→16	4→4	2→2
FH-tet-R	8→8	8→8	4→4
FH control	0.5→0.5	0.5→0.5	0.5→0.5

^*a*^ The symbol → indicates changes in the MICs when the antimicrobial drug is administered alone versus when it is combined with reserpine.

## DISCUSSION

In this study, we successfully selected tetracycline-resistant mutants through serial passages in subinhibitory concentrations of tetracycline and minocycline. Furthermore, several mutations in the 16S rRNA gene that correspond with changes in the MICs were identified.

Through serial passages in subinhibitory concentrations of tetracyclines, *M. pneumoniae* mutants demonstrating high-level resistance to tetracyclines (MIC ≥ 8 mg/L) were successfully generated. In the present study, 12 *M*. *pneumoniae* strains yielded eight resistant mutants following 14 passages. All the induced resistant mutants demonstrated cross-resistance to tetracycline and minocycline, while most remained sensitive to tigecycline. A study on macrolide-induced resistance in *M. pneumoniae* found that a resistant strain induced using midecamycin (a 16-membered macrolide) was resistant only to 16-membered macrolides but remained susceptible to 14- and 15-membered macrolides (25). This discrepancy may be attributed to different macrolides linked to mutations at different loci (25–27). Given the similarities in the resistance characteristics of the tetracycline- and minocycline-induced strains of *M. pneumoniae*, we hypothesize that their underlying resistance mechanisms are also similar. In addition, the remaining four induced strains exhibited increases in the MICs for all three tetracyclines regardless of whether they were induced with tetracycline, minocycline, or tigecycline. It follows that all *M. pneumoniae* strains have the potential to develop induced resistance when passaged continuously in an environment below the inhibitory concentration of tetracyclines. Although the resistance induction method used in this study is similar to that of Dégrange et al. (11), the difference in treatment concentrations and durations may explain why they did not obtain resistant mutants. Dégrange et al. performed only two rounds of passaging on *M. pneumoniae* parental strains under doxycycline concentrations at 2× and 8× MIC, ultimately obtaining mutant strains with the highest MIC for tetracycline at 2 mg/L. By contrast, our study refined the methodology established by Dégrange et al. by implementing a gradual stepwise escalation of antibiotic pressure. Following 12–14 consecutive passages under this regimen, we successfully isolated mutant strains exhibiting tetracycline MIC values ≥ 8 mg/L. These results demonstrate that a slow, incremental increase in antibiotic concentration during serial passaging facilitates the successful induction of tetracycline-resistant *M. pneumoniae* strains.

In addition, our experimental data demonstrate that tigecycline exhibits markedly lower propensity to induce resistance compared to tetracycline and minocycline, suggesting enhanced stability against resistance mechanisms. This may be because, unlike classical tetracyclines, tigecycline can evade efflux- and ribosome protection-mediated resistance mechanisms due to its distinctive structural characteristics (28). The emergence of each mechanism in human pathogens may be due to specific dynamics between tetracycline drug molecule structures and resistance mechanisms. Blake et al. demonstrated that each generation of tetracyclines preferentially selects for *Escherichia coli* expressing different resistance mechanisms (29). Tang et al. demonstrated that low-generation tetracyclines exhibited a greater propensity to stimulate the production and proliferation of antibiotic-resistant genes in the soil environment than did high-generation tigecyclines, an effect driven by the D-ring modifications found in high-generation tetracyclines (30). These findings strongly support prioritizing the use of third-generation tetracyclines in therapeutic regimens, especially when managing severe infections.

Bacterial resistance to tetracyclines is typically attributed to the horizontal exchange of resistance genes via mobile genetic elements, such as plasmids and transposons (31). According to the updated database of tetracycline resistance genes (https://faculty.washington.edu/marilynr/tetweb1.pdf), 75 tetracycline resistance genes and mosaic genes have been identified. However, despite the evidence linking high-level resistance to tetracyclines in *M. hominis* and *Ureaplasma* spp. to the presence of the *tetM* determinant (14, 15), whole-genome analyses of the tetracycline-resistant *M. pneumoniae* mutants induced in this study did not reveal any known acquired resistance genes. This aligns with the prevailing opinion that point mutations are the primary mechanism of antibiotic resistance in *M. pneumoniae* (4, 6, 25, 32, 33). It remains unclear whether acquired resistance genes are present in naturally occurring tetracycline-resistant *M. pneumoniae* strains. Despite the absence of detectable known tetracycline resistance determinants in the mutant strains, the concurrent lack of significant alterations in both efflux pump inhibitor efficacy and ribosomal drug-binding capacity observed in the S63-tet-R strain suggests the potential involvement of alternative resistance mechanisms, possibly mediated by unidentified genetic elements. For instance, chemical modifications in tetracycline-binding sites (34, 35), decreased outer membrane permeability due to aberrant expression of outer membrane proteins (36), and defective DNA repair (37) are associated with tetracycline resistance. However, systematic experimental validation is required to ascertain whether these mechanisms are responsible for *M. pneumoniae* resistance to tetracycline, and thus elucidate the underlying molecular basis of resistance.

Decreased ribosomal-binding capacity due to point mutations in the 16S rRNA gene is one of the mechanisms of tetracycline resistance in *M. pneumoniae*. We have identified three point mutations (T964C, G1169A, and A1173T) in the 16S rRNA gene that are associated with tetracycline resistance. The resistant mutants harboring T964C and G1169A mutations in the 16S rRNA exhibited lower levels of resistance to tetracyclines than those with the A1173T mutation. Dégrange et al. identified two point mutations (T968C and G1193A) in the 16S rRNA genes of the *M. pneumoniae* mutant with elevated MICs to tetracycline (11). Sulyok et al. found that mutations at positions 962–967, 1,058, 1,195, 1,196, and 1,199 in *Mycoplasma bovis* are associated with elevated tetracycline MICs (38). Base mutations in the 16S rRNA associated with tetracycline resistance have also been found in other bacteria, especially at positions AGA965 or A965, G966, and A967 in h31, G942 in h29, and G1058 in h34 (39–41). However, further investigations with more strains are needed to confirm whether mutations at a similar locus are responsible for reduced susceptibility or low levels of resistance to tetracyclines. The identification of mutations that confer resistance facilitates effective monitoring of tetracycline resistance in *M. pneumoniae* in a clinical setting and provides valuable insights into resistance breakpoints. In *M. pneumoniae*, mutations in the gene encoding for 23S rRNA, associated with macrolide binding, are the primary mechanism of resistance to macrolides (22, 42). In 1995, Lucier TS et al. demonstrated the effect of point mutations in the 23S rRNA gene of *M. pneumoniae* on macrolide binding using ^14^C-labeled erythromycin in a ribosome-binding assay (23). The T964C, G1169A, and A1173T point mutations identified in the tetracycline-resistant *M. pneumoniae* mutants in this study may lead to conformational changes that hamper base pairing and affect drug binding. Several studies have collected photoaffinity labeling or X-ray diffraction data to reveal the binding site of tetracyclines to bacterial 16S rRNA (43–45). These studies have confirmed that the tetracycline-binding pocket is constituted by several 16S rRNA secondary structure helices and their range of RNA residues. For instance, in *Thermus thermophilus*, the primary tetracycline-binding site is located in a pocket formed by residues 1,054–1,056 and 1,196–1,200 of H34, and 964–967 of H31, with several different secondary binding sites also being involved, including C1162-G1164 (H40) and G1172-G1174 (H40), among others (43). The mutations identified in this study have been shown to occur in, or in close proximity to, the tetracycline high-affinity binding sites on the ribosomal 30S subunit. We observed that resistant strains with mutations in the 16S rRNA gene exhibited reduced ability of ribosomes to bind tetracycline, thus confirming that decreased ribosome binding due to 16S rRNA point mutations may be the mechanism of tetracycline resistance in *M. pneumoniae*.

Efflux pumps also contribute to tetracycline resistance in *M. pneumoniae*. Bacterial resistance to antibiotics can be achieved via the active expulsion of intracellular antibiotics via transmembrane proteins. The efflux pumps for tetracycline antibiotics include those specific to tetracyclines, such as *tet*(*A-E*), *tetL*, and *tetK*, as well as a family of multidrug efflux pumps (46, 47). Reserpine inhibits the efflux activity of efflux pumps, including those belonging to the *tet* family and multidrug efflux pump family, thereby increasing bacterial susceptibility to antibiotics (48). Our study found that in the presence of reserpine, some tetracycline-resistant *M. pneumoniae* strains exhibited significantly reduced MICs of tetracyclines. This suggests that tetracycline resistance in *M. pneumoniae* is associated with efflux pumps. Nevertheless, no known efflux pump genes were identified. Further studies are warranted to validate whether novel, yet-to-be-characterized efflux pumps exist in *M. pneumoniae* that mediate tetracycline resistance.

The current study has some limitations. First, the *in vitro*-induced resistance assay does not account for the bacterial acquisition of resistance genes through the horizontal exchange of plasmids and transposons in clinical or natural settings and, therefore, does not fully represent the true clinical scenario. Continuous surveillance of *M. pneumoniae* susceptibility to tetracyclines in both clinical and environmental settings, combined with prompt mechanistic investigation of resistance trends, is essential to bridge this critical knowledge gap. Second, the stability of the resistance phenotype was not assessed. Third, due to experimental complexities, ribosome isolation and tetracycline-binding assays were performed on only two pairs of resistant mutants and susceptible parent strains, so the results may not be representative of all the resistant strains. Nevertheless, these limitations do not impact the findings of this study and its clinical value. In clinical practice, inappropriate antibiotic utilization significantly contributes to the development of resistance. Elucidating resistance mechanisms is critical for implementing rational antibiotic stewardship and devising targeted strategies to counteract antimicrobial resistance.

### Conclusions

In conclusion, the data show that *M. pneumoniae* can evolve to develop tetracycline resistance through long-term treatment with increasing concentrations of tetracyclines. Tigecycline is less effective in inducing resistance than tetracycline and minocycline. Mutations in the 16S rRNA gene leading to decreased ribosomal-binding capacity and the presence of efflux pumps may contribute to tetracycline resistance in *M. pneumoniae*. No *tetM* or other known tetracycline resistance genes could be identified in the resistant strains induced in this study. The findings of this study serve as a framework for monitoring the emergence of tetracycline resistance in *M. pneumoniae* to facilitate a more precise utilization of tetracyclines in clinical settings.
